# Repetition costs in sequence chunking

**DOI:** 10.3758/s13423-023-02338-7

**Published:** 2023-09-19

**Authors:** Rachel M. Brown, Iring Koch

**Affiliations:** Institute of Psychology, RWTH Aachen University, Jägerstraße 17-19, 52066 Aachen, Germany

**Keywords:** Sequence length, Action switching, Chunking, Action-effect compatibility, Sequence production

## Abstract

We examined how flexibly we plan sequences of actions when we switch between multiple action sequences. Mastering a sequential skill is assumed to involve integrating successive actions into groups known as chunks that can be efficiently planned and smoothly executed. Chunking is suggested by gains in planning efficiency for long compared to short action sequences following practice and learning associations between actions and perceptual outcomes. Less is understood about how efficiently we plan sequential chunks when we switch between multiple action sequences. Do we plan learned chunks less efficiently when we switch to a different action sequence? We examined this question by comparing the initiation and execution latencies of long versus short action sequences, performed from memory, when sequences switched or repeated across trials. Additionally, each action within the sequences generated predictable perceptual outcomes that were either spatially compatible or spatially incompatible with the action sequences. Results suggested repetition costs (instead of benefits) when performing long sequences. Repetition, as opposed to switching, prolonged initiation and increased the error rate of long compared to short sequences. We attribute these results to the flexible coordination of chunk planning and execution. Repetition may prolong advanced planning of long sequences in order to resolve conflict between multiple chunks, and switching may allow the planning of later chunks to be postponed until execution. We propose that the chunking of action sequences can both facilitate and interfere with action-switching performance.

## Introduction

We have the remarkable ability to learn complex series of temporally ordered actions, by grouping successive actions into integrated units, or chunks (Klapp, 1995; Lashley, 1951; Sakai et al., 2003; Verwey & Eikelboom, 2003). When we learn to drive or type, we tend to be slow and inaccurate at first, but with practice we press the clutch then shift the gear, or type letters in a word, all in one fluid and accurate motion. When we practice a new skill, it is thought that we initially retrieve and execute sequential actions one-by-one (Doyon & Benali, 2005; Wymbs et al., 2012), but eventually we learn to integrate series of once-distinct movements into tightly associated chunks (Fitts & Posner, 1967; Verwey & Abrahamse, 2012). Each chunk can then be retrieved from memory and executed as a single unit (Abrahamse et al., 2013; Fitts & Posner, 1967; Keele, 1968; Klapp, 1995; Logan et al., 2011; Logan & Crump, 2011; Ramkumar et al., 2016; Summers & Anson, 2009; Verwey & Eikelboom, 2003). The question remains, once we learn sequential skills, can we plan sequences of actions in a flexible way?

Voluntary actions are assumed to be controlled by two distinct processes: an “outer-loop” or cognitive processor that selects or retrieves the appropriate action and prepares it for execution (we refer to these steps collectively as “planning”), and an “inner-loop” or motor processor that executes the prepared action (“dual-processor” view, Abrahamse et al., 2013; “two-loop theory,” Logan & Crump, 2011). The chunking of learned sequential actions is assumed to allow an additional level of hierarchical control, where actions within a chunk are planned and executed efficiently as a single unit (Diedrichsen & Kornysheva, 2015; Logan & Crump, 2011; Verwey et al., 2015). Two observations align with this view. First, longer sequences often take longer to initiate (i.e., to begin the first action in the sequence) compared to shorter sequences (Garcia-Colera & Semjen, 1987; Klapp, 1995; Sternberg et al., 1978; Stöcker & Hoffmann, 2004; Verwey, 2003; Verwey & Eikelboom, 2003), an observation known as the *sequence length* effect. This observation is assumed to reflect the additional time required to plan more items prior to execution (Sternberg et al., 1978; Verwey & Eikelboom, 2003). Second, the sequence length effect typically decreases with practice (Klapp, 1995; Stöcker & Hoffmann, 2004; Verwey, 2003; Verwey & Eikelboom, 2003). This observation is attributed to chunking, which allows fewer units to be planned before execution. For instance, experienced typists were slower to type the first letter in long *non-words* (nonsense words) compared to short non-words, but they typed the first letter in long and short *words* with similar latency (Yamaguchi & Logan, 2014), suggesting that letters within words could be planned together as integrated units.

A key assumption in sequential action control is that chunking can ease the cognitive burden on sequence planning (Diedrichsen & Kornysheva, 2015; Verwey et al., 2015). This assumption naturally invites the question: how flexible is sequence planning when we switch between multiple learned (chunked) sequential actions? Action switching is ubiquitous in daily goal-directed behavior, such as alternating between shifting gears and steering when driving. It is well documented that switching between different goal-directed behaviors (i.e., “tasks”) entails slower and less accurate performance compared to repeating the same task, an observation known as a *switch cost* (for reviews, see Koch et al., 2018; Monsell, 2003). Much of the evidence for switch costs comes from single-response tasks (e.g., pressing a button) (Koch et al., 2018), although some recent work has also demonstrated switch costs for short (e.g., three-element) sequential actions (Brown et al., 2022; Rieger et al., 2017). It is not yet known how action switching impacts the planning efficiency of longer sequences relative to that of shorter sequences. Switch costs are thought to reflect extra time needed to overcome cognitive constraints when retrieving the current task, such as capacity limitations or the persistence of a previous task in memory (Gade et al., 2014; Kiesel et al., 2010; Pashler, 1994; Vandierendonck et al., 2010). According to this view, switching should place more demands on these cognitive control processes when planning longer sequences. Alternatively, chunking may help overcome the demands of switching, such that long and short sequences can be planned with similar efficiency when they switch or repeat. The former prediction implies that action planning efficiency is context-dependent, while the latter prediction implies that chunking allows stable planning efficiency across different contexts.

Chunking may also be aided by associating actions with their perceptual outcomes (“action effects”) (Rieger, 2004; Snyder et al., 2015). From an ideomotor perspective, planning movements depends on first anticipating the movement’s desired effects (Greenwald, 1970; James, 1890; Shin et al., 2010), such as which letters you want to appear when typing. When effects are fully predictable based on the preceding action (pressing the “A” key always makes “A” appear), the anticipated effect should prime the action (Land, 2018; Thomaschke et al., 2018). Thus, sequential action planning should be facilitated when perceivable effects predictably follow each action. Accordingly, the sequence length effect decreased over practice when the actions in each sequence consistently generated distinct tones as opposed to no tones or uniform tones (Stöcker & Hoffmann, 2004), possibly because anticipating the predictable tones speeded retrieval of the correct sequence. This finding suggests that chunking may be facilitated by the *compatibility*, or the degree of feature overlap, between actions and their predictable effects (e.g., Greenwald, 1972). For instance, actions and effects are spatially compatible when they occur in corresponding spatial locations (e.g., a left button press generates a stimulus on the left), in contrast to incompatible actions and effects (e.g., a left button press generates a stimulus on the right). Both single-response and sequential actions are speeded when they predictably generate spatially compatible effects compared to incompatible effects (Greenwald, 1972; Keller & Koch, 2006, 2008; Koch et al., 2004; Kunde, 2001; Kunde et al., 2004; Pfister, 2019), suggesting that compatible effects could be easy to anticipate *prior* to performing the action, which may in turn help retrieve the correct action. The question remains, does action-effect compatibility increase sequential planning efficiency by facilitating chunking? If so, spatially compatible action effects should reduce the sequence length effect.

The primary goal of the current study was to examine how action switching influences the relative planning efficiency of long and short sequences, in order to infer how action switching influences the planning of sequential chunks. The secondary goal was to examine the influence of spatial action-effect compatibility on planning efficiency. Participants first learned a short and a long action sequence. They then performed the sequences from memory, during which the action sequences either switched or repeated across trials, and each action generated spatially compatible or incompatible visual effects. We examined the latencies of sequence initiation and each sequential response, as well as error rates. We predicted, based on classical switch costs, that switching compared to repeating action sequences would increase the sequence length effect (greater initiation latency for long compared to short sequences), and that compatible (as opposed to incompatible) action effects would reduce the sequence length effect.

## Method

### Participants

Forty-two volunteers participated in the study via Prolific (prolific.co). Pre-screening criteria limited online recruitment to individuals who had upon registration with Prolific declared themselves to be between the ages of 18 and 35 years, right-handed, and to have normal or corrected-to-normal vision. After completing the study, participants were asked to verify their age, handedness, vision correction, and if they were neurologically healthy. Two participants were excluded because they did not report that they were neurologically healthy. This yielded a final sample of 40 participants, which included 15 females and a mean age of 24.8 years (*SD* = 5.5, range 18–35 years). Participants provided informed consent, and the study was approved by the internal ethics committee at the Institute of Psychology at RWTH Aachen University and was conducted according to tenets of the Declaration of Helsinki.

A sample size of at least *N* = 30 per experiment was estimated based on an a priori power analysis and two pilot experiments (see Appendix). Because theoretical perspectives on chunking, task switching, and action-effect compatibility do not make explicit assumptions about effect sizes, we instead estimated a minimum effect size of *η*^*2*^_*p*_ = .32 based on previously reported sequence length effects [*η*^*2*^_*p*_ = .38, *η*^*2*^_*p*_ = .32, *η*^*2*^_*p*_ = .33] (Garcia-Colera & Semjen, 1987; Stöcker & Hoffmann, 2004; Verwey & Eikelboom, 2003), previously reported effects of action sequence switching on sequence initiation latency [*η*^*2*^_*p*_ = .52] (Brown et al., 2022), and previously reported effects of visual-spatial action-effect compatibility on action latency [*η*^*2*^_*p*_ = .53] (Kunde, 2001), all within-subjects manipulations of the factor in question. Power analysis using an alpha level of *α* = .05 and a power level of 1 – *β* = .80 suggested a sample size of *N* = 29, which we increased to at least 30 per group in two pilot experiments (see Appendix) and to *N* = 40 in the main experiment (yielding an achieved power of .93).

### Equipment and stimuli

**Fig. 1:**
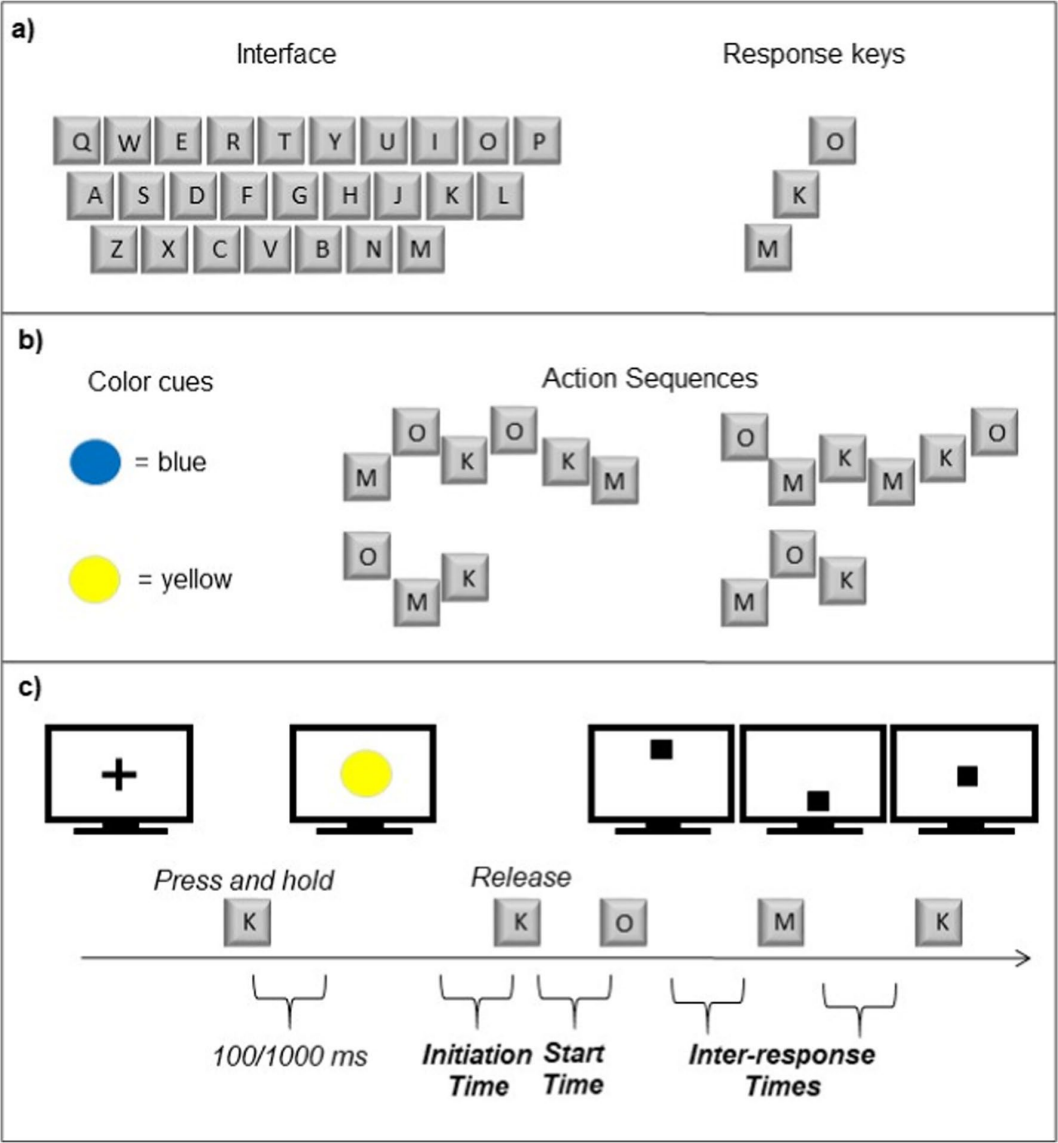
Interface, sequences, stimuli, and trial procedure. **a**) The top panel shows an example keyboard interface, and the keys used to perform the sequences (note that the same keys could be used on a QWERTY keyboard shown here, or a QWERTZ keyboard). It was not possible to control or check whether participants used a different keyboard layout. If some participants used a non-standard keyboard layout, this would have lessened or negated the compatibility effect for those participants, resulting in underestimated compatibility effects. **b**) The middle panel shows which colors were used to cue each sequence, and it shows the corresponding action sequences. **c**) The lower panel illustrates the progression of a trial within the task (here a trial with a short sequence with compatible action effects)

The experiment was conducted online using Gorilla (gorilla.sc). Participants were required to complete the study on their own laptop or desktop computer. Participants pressed the “M”, “K”, and “O” keys with their index, middle, and ring finger, respectively, of their right hand (Fig. 1a), in four possible sequential orders: two possible long sequences (M-O-K-O-K-M, or O-M-K-M-K-O) or two possible short sequences (O-M-K, or M-O-K) (factor “Length”) (Fig. 1b). Half of the participants performed M-O-K-O-K-M and O-M-K, and the other half performed O-M-K-M-K-O and M-O-K. Thus, each participant performed one short and one long sequence, which we modelled on previous sequence-length effect paradigms to ensure that our task was of comparable difficulty (Stöcker & Hoffmann, 2004; Verwey, 2003; Verwey & Eikelboom, 2003).

At the beginning of each trial a fixation cross was presented in the center of the screen, followed by one of two possible colored circles, one yellow and one blue, approximately 5 cm in diameter, which served as imperative cues signifying the correct sequence of key-presses to be performed. A blue circle indicated the long sequence, and a yellow circle indicated the short sequence (Fig. 1b). When participants first learned the sequences (learning task), the digits “1”, “2”, or “3” appeared one at a time below the circle. These digits functioned as response cues, to prompt participants to press the “M”, “K”, or “O” keys, respectively. Once the participants learned the sequences, they performed them from memory following the presentation of a color cue. Additionally, each key-press resulted in the appearance of a black square in one of three possible positions in a vertical array along the center of the screen (see Fig. 1c), mirroring the (approximate) vertical alignment of the response keys. The squares were approximately 3 cm in height and were spaced about 4 mm apart.

### Procedure

#### Learning and practice tasks

The participants first learned the key-press sequences and their corresponding color cues by performing a serial response-time task (learning task). Each trial began with a fixation cross presented for 1,500 milliseconds (ms), followed by the appearance of a blue or yellow colored circle. After 800 ms, the digit “1”, “2”, or “3” was presented on the screen below the colored circle and remained on the screen until the participant pressed the correct key. Each correct key-press elicited the presentation of another digit corresponding to the next key-press in the sequence, until the sequence was completed. The temporal interval between each correct key-press and the presentation of the next digit was set to 800 ms, in order to encourage participants to perform accurately and not too quickly (Stöcker & Hoffmann, 2004). Any incorrect key-press elicited an error feedback message (“Error”) presented for 1,000 ms, and ended the trial. Otherwise, a correctly performed trial ended with a “Correct!” feedback message presented for 500 ms. The colored circle remained on the screen until the sequence was correctly completed, or until an incorrect key-press was made (the circle disappeared before either feedback message was presented). The learning task comprised three blocks of 28 trials each, such that each block included 14 presentations each of the long and short sequence. In the third and final block, the digits “1”, “2”, and “3” were replaced with a question mark (“?”), which remained on the screen until the participant responded with the correct key-press. This was done to allow participants to start to practice performing the sequences from memory. All participants performed the learning task with 85% or greater accuracy (*M* = 98.18%, *SD* = 2.67).

After completing the learning task, participants completed a brief practice task to ensure that they could perform the sequences accurately from memory, and to practice the format of the upcoming main task of the experiment. Participants were required to perform each sequence correctly from memory three times in total in order to continue to the main task. Each trial began with a fixation cross, which remained on the screen until participants made the following response: participants were to continue the trial by *pressing and holding down the “K” key*. While the participant held down the “K” key, one of the two color cues (a blue or yellow circle) appeared after an interval of either 100 or 1,000 ms (pseudo-randomly varied across trials). This interval was varied because, as reported previously, when this interval was predictable participants tended to respond with temporal regularity rather than speed (see Keller & Koch, 2008). The cue remained on the screen until the participant *released the “K” key*. After releasing the “K” key, the participant then performed the sequence of key-presses. Participants were instructed, upon appearance of the color cue, to both release the “K” key and subsequently perform the sequence as quickly and accurately as possible. If participants made any incorrect response within the sequence, the trial ended and an error feedback message appeared (“Error”) for 1,000 ms, and the participants were then required to repeat the trial. If participants performed the sequence correctly, a “Correct!” feedback message appeared for 1,000 ms. Thus, if participants made no errors, they performed each sequence three times in a pseudorandom order. Each incorrect response resulted in a repeated trial. Participants performed each sequence correctly three times in succession after a mean of 4.15 trials (*SD* = 1.58, max = 9).

#### Main task: Performing from memory

Participants then completed the main task of the experiment, which involved performing the sequences from memory. The procedure during each trial was identical to that of the practice task, except that now each key-press response elicited the presentation of action effects. Each trial began with a fixation cross, which remained on the screen until participants pressed and held down the “K” key. While the participant held down the “K” key, one of the two color cues (a blue or yellow circle) appeared after either 100 or 1,000 ms (pseudo-randomly varied across trials). The cue remained on the screen until the participant released the “K” key, after which they performed the key-press sequence. Participants were instructed to perform as quickly and accurately as they could. Any incorrect key-press elicited an error feedback message (“Error”) presented for 1,000 ms, and ended the trial. Each correct key-press immediately elicited an action effect: a black square that appeared in either the upper-center, middle-center, or lower-center of the screen (see Fig. 1c). Each black square remained on the screen until the next key-press in the sequence was made, except for the final square, which remained on the screen for 500 ms.

The entire task consisted of eight blocks of 28 trials each, such that each block included 14 presentations each of the long-sequence cue and the short-sequence cue. Within each block, the number of action switches and action repetitions from trial to trial was equalized (factor “Switch”). The correspondence between each possible key-press and each possible action effect was either spatially compatible or spatially incompatible (factor “Compatibility”). In the case of spatially compatible action effects, the spatial contour of the action effect sequence followed the same spatial contour of the key-press sequence: the top-most key (“O”) elicited the top-most square on the screen, the middle key (“K”) elicited the middle square, and the bottom-most key (“M”) elicited the bottom-most square on the screen (top-middle-bottom keys → top-middle-bottom effects). In the case of spatially incompatible action effects, the spatial contour of the action effect sequence did not follow the same spatial contour of the key-press sequence. Two possible incompatible action-to-effect mappings were used: (1) top-middle-bottom keys → bottom-top-middle effects, or (2) top-middle-bottom keys →  middle-bottom-top effects. The two incompatible mappings were counterbalanced across participants, such that each participant was presented with only one incompatible mapping during the entire experiment. The eight task blocks always included four consecutive blocks of compatible action effects and four consecutive blocks of incompatible action effects, so that compatibility was consistent across consecutive trials, and action effects were always fully predictable. The order in which compatible and incompatible blocks occurred during the experiment (whether the compatible blocks preceded the incompatible blocks or vice versa) was counterbalanced across participants. The entire experiment lasted between 30 and 45 min.

### Design and analyses

The design consisted of a 2 × 2 × 2 within-subjects factor structure with factors Length (short or long sequential action), Switch (switch or repetition), and Compatibility (compatible or incompatible action effects). Four dependent measures, three response-time measures and error rate, were calculated from the responses recorded during the main task. To examine sequence length effects, we examined “initiation time,” which was the time in milliseconds from the onset of the color cue (the colored circle) to the onset of the participant’s release of the “K” key (see Fig. 1c). We interpret initiation time to include the time required to retrieve the correct action sequence and to prepare and initiate the action (see Keller & Koch, 2008). We also examined “start time,” which was the time in milliseconds from the onset of the participant’s release of the “K” key to the onset of the first key-press in the sequence (see Fig. 1c). We additionally examined “inter-response time,” which was the response time in milliseconds for each of the individual key-presses in the sequence other than the first key-press (the time from the onset of a previous key-press in the sequence to the onset of the current key-press, for key-presses 2 and 3 in the short sequence, or key-presses 2, 3, 4, 5, and 6 in the long sequence; see Fig. 1c). The mean of all inter-response times in a sequence was interpreted as the mean time required to execute an individual response in the sequence. Finally, “error rate” was calculated as the percentage of trials in which the sequence was performed incorrectly. The first trial of each block was excluded from the above calculations, and all trials in which initiation time, start time, or inter-response time differed from their respective means (excluding trial 1 of each block) by more than three standard deviations were excluded from further analysis. All response time variables additionally excluded trials in which the sequence was performed incorrectly and all trials immediately following incorrect sequence performance.

For each dependent variable, a within-subjects ANOVA with the described 2 × 2 × 2 factor structure was run. Results from each ANOVA are reported in Table 1, and marginal/cell means are reported in Table 2 with associated standard error and 95% confidence intervals. Post hoc test *p* values were adjusted for familywise comparisons using the Tukey method. In addition to partial and generalized *η*^*2*^, we report *Hedges’s g*_*rm*_ for pairwise comparisons (notated as “*g*_*rm*_”), which is a variation of Cohen’s *d* for correlated samples that gives a less biased estimation of population effect size (Lakens, 2013). Data processing and statistical analyses were performed in R version 4.1.3 using the afex and emmeans packages.

**Table 1:** ANOVA summary table per dependent variable

Initiation time	Source	*df*	MSE	*F*	*η*^*2*^_*G*_	*η*^*2*^_*p*_	*p*
	Length	1, 39	1694.82	13.31	.003	.25	<.001
	Switch	1, 39	4554.08	0.23	<.001	.01	.631
	Compatibility	1, 39	12824.84	0.30	<.001	.01	.584
	Length × Switch	1, 39	2770.21	14.02	.005	.26	<.001
	Length × Compatibility	1, 39	733.22	1.37	<.001	.03	.249
	Switch × Compatibility	1, 39	1734.52	0.02	<.001	<.01	.888
	Length × Switch × Compatibility	1, 39	1024.11	4.26	<.001	.10	.046
Start time	Source	*df*	MSE	*F*	*η*^*2*^_*G*_	*η*^*2*^_*p*_	*p*
	Length	1, 39	6969.25	11.97	.009	.23	.001
	Switch	1, 39	5389.70	2.78	.002	.07	.104
	Compatibility	1, 39	14246.88	0.31	<.001	.01	.580
	Length × Switch	1, 39	6862.93	18.51	.014	.32	<.001
	Length × Compatibility	1, 39	1878.22	1.15	<.001	.03	.291
	Switch × Compatibility	1, 39	2411.44	3.39	<.001	.08	.073
	Length × Switch × Compatibility	1, 39	2179.24	1.35	<.001	.03	.252
Inter-response time	Source	*df*	MSE	*F*	*η*^*2*^_*G*_	*η*^*2*^_*p*_	*p*
	Length	1, 39	804.73	68.58	.037	.64	<.001
	Switch	1, 39	145.08	0.03	<.001	<.01	.867
	Compatibility	1, 39	1884.14	0.70	<.001	.02	.408
	Length × Switch	1, 39	143.53	0.04	<.001	<.01	.844
	Length × Compatibility	1, 39	171.40	2.29	<.001	.06	.138
	Switch × Compatibility	1, 39	62.88	0.18	<.001	<.01	.678
	Length × Switch × Compatibility	1, 39	94.62	0.20	<.001	<.01	.658
Error rate	Source	*df*	MSE	*F*	*η*^*2*^_*G*_	*η*^*2*^_*p*_	*p*
	Length	1, 39	76.18	11.28	.038	.22	.002
	Switch	1, 39	68.11	2.19	.007	.05	.147
	Compatibility	1, 39	37.29	1.78	.003	.04	.190
	Length × Switch	1, 39	53.99	4.52	.011	.10	.040
	Length × Compatibility	1, 39	26.57	4.48	.005	.10	.041
	Switch × Compatibility	1, 39	30.19	0.13	<.001	<.01	.722
	Length × Switch × Compatibility	1, 39	21.54	0.20	<.001	.01	.658

*MSE* mean square error

**Table 2:** Pairwise comparisons per dependent variable

	Long		Short		difference	*t(39)*	*p*	*g*_*rm*_
	M (SE)	CI	M (SE)	CI	M (SE)			
Initiation time (ms)								
Incompatible								
Repeat	550 (23.7)	[502 598]	501 (19.1)	[462 539]	49.8 (9.9)	5.05	< .001	.32
Switch	524 (25.1)	[473 575]	533 (25.9)	[481 585]	9.1 (8.7)	1.04	.964	.06
Compatible								
Repeat	532 (24.0)	[483 580]	504 (20.6)	[462 545]	27.9 (8.6)	3.26	.043	.18
Switch	521 (28.0)	[465 578]	523 (29.0)	[464 582]	1.4 (8.1)	0.17	>.999	.01
Start time (ms)								
Repeat	288 (28.6)	[230 346]	216 (18.2)	[179 253]	72.1 (16.4)	4.41	< .001	.38
Switch	262 (27.4)	[206 317]	269 (27.0)	[215 324]	7.6 (8.9)	0.85	.829	.04
Inter-response time (ms)	236 (9.8)	[216 256]	210 (10.8)	[188 231]				
Error rate (%)								
Repeat	11.0 (1.5)	[7.9 14.1]	6.0 (1.2)	[3.6 8.3]	5.02 (1.41)	3.56	.005	.57
Switch	7.9 (1.2)	[5.6 10.2]	6.4 (0.8)	[4.7 8.0]	1.53 (1.12)	1.37	.528	.24
Incompatible	8.4 (1.2)	[6.1 10.7]	6.3 (0.9)	[4.5 8.1]	2.1 (1.0)	2.1	.177	.31
Compatible	10.5 (1.5)	[7.5 13.5]	6.0 (0.7)	[4.5 7.5]	4.5 (1.3)	3.6	.005	.54

*g*_*rm*_ *Hedges’s* g_rm_, *M* mean, *SE* standard error of the mean; *CI* 95% confidence interval

## Results

### Initiation time

**Fig. 2:**
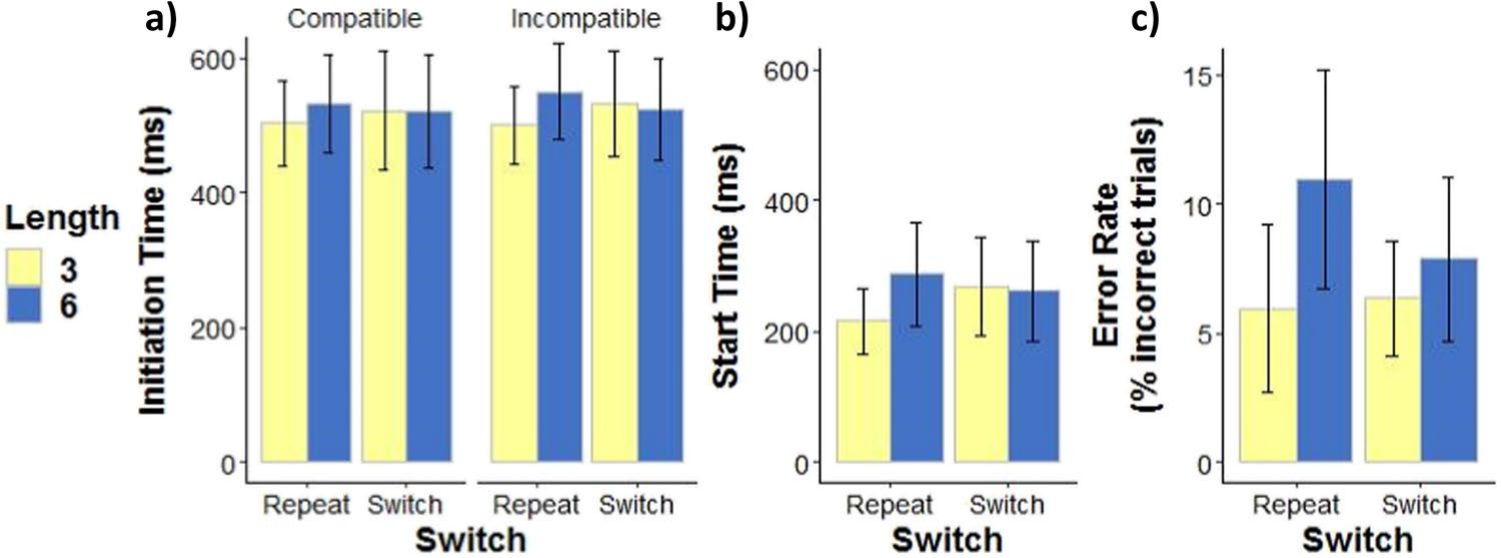
Initiation time, Start time, and Error rate. **a**) Effects of Length (6 = long sequence, 3 = short sequence), Switch (Repeat = sequence repetition, Switch = sequence switch), and Compatibility (Compatible = spatially compatible action effects, Incompatible = spatially incompatible action effects) on initiation time (ms = milliseconds). **b**) Effects of Length and Switch on start time. **c**) Effects of Length and Switch on error rate (percent incorrect trials). In all panels (**a**, **b**, and **c**) marginal means are plotted and error bars represent 95% confidence intervals

A main effect of Length, a Length-by-Switch interaction, and a Length-by-Switch-by-Compatibility interaction (see Table 1) indicated longer initiation times for long sequences compared to short sequences in Repeat but not Switch trials (Length-by-Switch interaction), and this pattern was more pronounced in the incompatible condition compared to the compatible condition (three-way interaction; see Fig. 2a). In Repeat trials, initiation time (ms) for long sequences exceeded that of short sequences, and this difference was greater in the incompatible condition compared to the compatible condition (see Table 2). In Switch trials, the difference between long and short sequences was relatively small in both the incompatible condition and in the compatible condition (Table 2). A pilot experiment also found marginally greater initiation time for long sequences in Repeat trials (a marginal Length-by-Switch interaction; see Appendix Table 3, Fig. 4). Thus, repeating a long sequence prolonged initiation time, particularly when actions and effects were incompatible.

### Start time

A main effect of Length and a Length-by-Switch interaction (see Table 1) indicated longer start times for long sequences compared to short sequences in Repeat but not Switch trials (see Fig. 2b), mirroring the initiation time results (above). In Repeat trials, start time (ms) for long sequences exceeded that of short sequences (see Table 2). In Switch trials, the difference between long and short sequences was relatively small (Table 2). This result was replicated in both pilot experiments (see Appendix Tables 3 and 4 and Fig. 4).

### Mean inter-response time

A main effect of Length (see Table 1) indicated longer mean inter-response time (ms) for long sequences compared to short sequences (see Table 2). This result was replicated in both pilot experiments (see Appendix Tables 3 and 4).

### Error rate

A main effect of Length and a Length-by-Switch interaction (see Table 1) indicated higher error rate in long sequences compared to short sequences, and this difference was greater in Repeat trials compared to Switch trials (see Fig. 2c), mirroring the initiation time and start time results (above). In Repeat trials, error rate (percent incorrect trials) for long sequences exceeded that of short sequences (see Table 2). In Switch trials, the difference between long and short sequences was smaller (Table 2).

In addition, a Length-by-Compatibility interaction (Table 1) indicated higher error rate for long sequences compared to short sequences in the compatible condition, and this difference was smaller in the incompatible condition (see Table 2).

### Inter-response time chunking

**Fig. 3:**
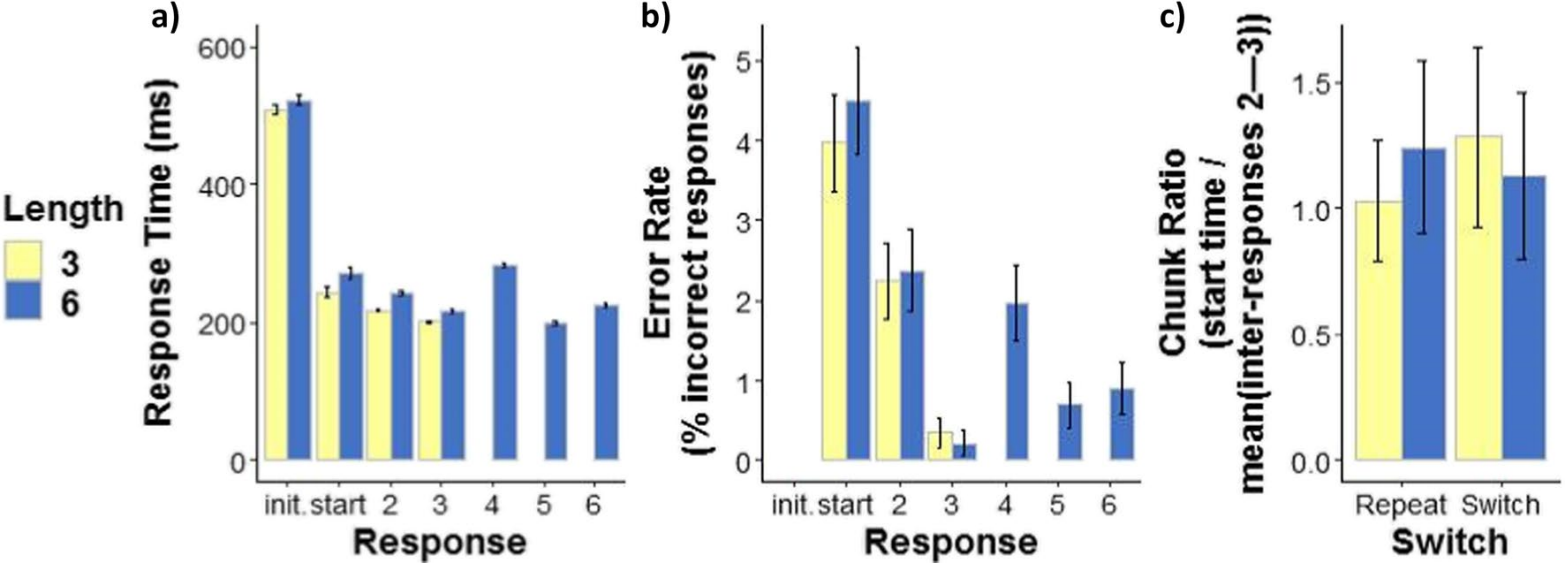
Inter-response time chunking: Inter-response times, Error rates, and Chunk ratios. **a**) Mean response latencies for all responses in long and short sequences (6 = long sequence, 3 = short sequence) (init. = initiation time, start = start time, numbers 2–6 = inter-responses 2 through 6 in long sequences, numbers 2–3 = inter-responses 2 through 3 in short sequences) (ms = milliseconds). **b**) Error rate (percent incorrect responses) for all responses (note that initiation is not included because it was always performed correctly) for long and short sequences. **c**) Effect of Length (6 = first half of the long sequence, 3 = short sequence) and Switch (Repeat = sequence repetition, Switch = sequence switch) on mean chunk ratio (start time divided by the mean of inter-response times 2 and 3). Here, marginal means are plotted. In all panels (**a**, **b**, and **c**) error bars represent 95% confidence intervals

**Fig. 4:**
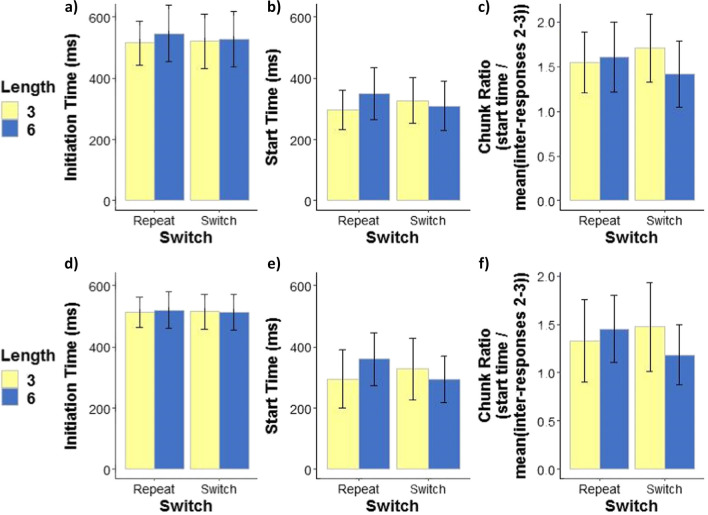
Pilot Experiments 1 and 2: Initiation time, Start time, and Chunk ratio. **a**) Pilot Experiment 1: Effects of Length (6 = long sequence, 3 = short sequence) and Switch (Repeat = sequence repetition, Switch = sequence switch) on initiation time (ms = milliseconds). **b**) Pilot Experiment 1: Effects of Length and Switch on start time. **c**) Pilot Experiment 1: Effects of Length and Switch on chunk ratio (start time divided by the mean of inter-responses 2–3). **d**) Pilot Experiment 2: Effects of Length and Switch on initiation time. **e**) Pilot Experiment 2: Effects of Length and Switch on start time. **f**) Pilot Experiment 2: Effects of Length and Switch on chunk ratio. In all panels (**a**, **b**, **c**, **d**, **e**, and **f**) marginal means are plotted and error bars represent 95% confidence intervals

To examine how participants chunked the long sequence, visual inspection of each response indicated longer inter-response time and higher error rate on the *fourth* response in the long sequence (see Fig. 3a and b), suggesting that participants chunked the long sequence into two halves (Bo & Seidler, 2009; Sakai et al., 2004; Verwey & Eikelboom, 2003). We then compared the first half of the long sequence to the short sequence (both equal in length) by calculating the ratio of the start time to the mean of inter-responses 2 and 3 (response times within a chunk) in both long and short sequences. Larger “chunk” ratios are assumed to indicate response time speeding due to sequential priming within a chunk (Bo & Seidler, 2009; Verwey & Eikelboom, 2003). A 2 × 2 × 2 ANOVA on chunk ratios (with the same fixed-factor structure) indicated a Length-by-Switch interaction (*F*(1,39) = 18.57, *MSE* = 0.15, *p* < .001, *η*^*2*^_*G*_ = .015, *η*^*2*^_*p*_ = .32). In Switch trials, the chunk ratio of short sequences exceeded that of long sequences (*short*: *M =* 1.28*, SE =* 0.129*,* 95%CI = [1.024, 1.55]; *long*: *M =* 1.13*, SE =* 0.118*,* 95%CI = [0.891, 1.37]; *difference* = 0.155, *SE =* 0.0416, *t(39)=* 3.717, *p* = .0034, *g*_*rm*_* =* .18) – this result was also found in both pilot experiments (see Appendix Results). In Repeat trials, the chunk ratio of long sequences exceeded that of short sequences (*long*: *M =* 1.24*, SE =* 0.124*,* 95%CI = [0.993, 1.49]; *short*: *M =* 1.03*, SE =* 0.087*,* 95%CI = [0.853, 1.21]; *difference* = 0.214, *SE =* 0.068, *t(39)=* 3.144, *p* = .0161, *g*_*rm*_* =* .27) (see Fig. 3c). No other statistically significant effects were found. These results suggest that participants executed the first half of the long sequence more slowly than the short sequence when switching, but they executed the first half of the long sequence more quickly than the short sequence when repeating the same sequence.

## Discussion

### Summary of main findings

The first goal of the current study was to examine whether switching from one action sequence to another lengthens the time needed to plan longer (compared to shorter) sequences in advance of execution (increased sequence length effect). Contrary to this prediction, we observed that sequence *repetition* increased the sequence length effect, compared to switching. This result was consistent in both response time and error measures, and it was replicated in two pilot experiments (see Appendix), attesting to its reliability. When participants repeated long sequences: (1) they took longer to initiate the sequence, (2) they took longer to make the first response in the sequence, and (3) they made more errors, all in comparison to repeating short sequences. No differences in the above response latencies or error rates were detected between long and short sequences when switching. The pattern of inter-response times suggested that participants chunked the long sequence into two units. When participants switched action sequences, they were relatively slower to execute elements within the first chunk of the long sequences. These results suggest that repetition may prolong *advanced planning* and switching may prolong *execution* of long sequences.

The second goal was to examine whether action-effect compatibility speeds advanced planning (reduces the sequence length effect). In line with this prediction, spatially compatible (compared to incompatible) action effects *reduced* the sequence length effect but specifically during sequence repetition. We suggest that anticipating the compatible effects may have helped overcome the increased planning demands of repeating long sequences.

### Action sequence switching and sequence length

Participants appeared to be slower and less accurate when planning longer sequences that they had just performed. This result implies that repetition imposes efficiency and accuracy costs on long relative to short sequences. On the one hand, this finding is surprising given that an action that has just been performed may linger in working memory (Gade et al., 2014; Kiesel et al., 2010; Klapp, 1995; Vandierendonck et al., 2010), and should therefore be easier to plan. On the other hand, the finding could be explained if we assume that (1) the long sequence was chunked into multiple units, and (2) only the final chunk remained available in working memory after the long sequence was just performed (a recency effect). This final chunk may be a source of conflict, because it is incorrect as a starting chunk, but it is still relevant to (a part of) the current sequence to be performed. This scenario may be similar to a “partial repetition cost” in a task-switching paradigm: people are typically slower and less accurate when some but not all features of the previous task carry over to the current task, compared to when all features of the current task are different from the previous task (Brown et al., 2022; Schacherer & Hazeltine, 2022; Weissman et al., 2023). In the current study, additional planning time may have been needed to resolve an inter-chunk conflict, possibly because the still-relevant final chunk is difficult to inhibit while retrieving the first (correct) chunk (for reviews, see Koch et al., 2018; Monsell, 2003). In contrast, when switching to a different sequence (whether long or short), a lingering memory trace of the previous sequence should create comparatively less conflict, because the previous and current sequences are independent. This interpretation aligns with the possibility that there are general constraints on the number or size of chunks that can be maintained in working memory (see, e.g., Klapp, 1995; Logan et al., 2011; Palmer & Pfordresher, 2003). Both response latencies and error rates were highest overall for long, repeated sequences, suggesting that repetition did not simply speed the short sequences, but instead may have interfered with accurate retrieval of the long sequence. Thus, we hypothesize that if the long sequences were encoded as two distinct planning units (chunks), repeating the long sequence may have required a “partial repetition” from one chunk to another.

An additional explanation for the results is that switching was beneficial for planning long sequences, because planning the second chunk could be postponed until sequence execution. Participants may have shown lower chunk ratios in the long (compared to short) sequences during switch trials because they executed the first chunk more slowly in order to allow time to plan the second chunk. In this case participants would only need to retrieve the first chunk of the long sequence before execution, which would explain the similar pre-execution latencies (initiation and start times) for short and long sequences when switching. This interpretation aligns with the notion of independent processors for planning and executing action sequences (Abrahamse et al., 2013; Logan & Crump, 2011). These findings further suggest that the two processors may work together flexibly, dynamically adjusting whether the current sequence is planned in advance or incrementally, based on current constraints.

In general, slower and less accurate performance when repeating compared to switching an action could resemble inhibition of return (IOR), or a tendency for attention to be diverted away from a recently attended task or object. IOR effects are often observed in tasks that involve responding to sudden spatial stimuli (Dukewich, 2009; Klein, 2000; Lupiáñez et al., 2006). Interestingly, recent work suggests an IOR-type effect when retrieving recently read words (Langerock et al., 2021). This work raises the possibility that phenomena similar to IOR might occur when performing motor sequences from memory. Here we observed that repetition was detrimental to longer compared to shorter sequences, which would speak to a length-specific rather than a general IOR effect. Further work could examine whether and how IOR phenomena occur in sequence production.

The findings here document an influence of trial-to-trial transitions on the sequence length effect. Note that the current results cannot specify whether this influence was due to switching to a different sequence or switching to a different sequence length, because all switch trials were also length switches. The results here nonetheless show that the sequence length effect changes as a function of repetition versus switching in general, which could be due to a switch of the length or sequence identity dimension, or both. This suggests that sequence planning may flexibly adjust to the cognitive demands imposed by the current transition. Previous work has documented sequence length effects across different paradigms in which short and long sequences are either interleaved (Stöcker & Hoffmann, 2004; Verwey, 2003; Verwey & Eikelboom, 2003; Yamaguchi & Logan, 2014) or blocked (Garcia-Colera & Semjen, 1987; Klapp, 1995), suggesting that the effect on average may not necessarily depend on switching or repetition. Rather, the results reported here may help shed light on the trial-to-trial dynamics of the sequence length effect.

It is also important to note that the results reported here may not reflect highly practiced sequential performance, such as typing a word (Yamaguchi & Logan, 2014). Although participants here showed relatively high accuracy, they may not have had enough practice (42 learning trials per sequence) to achieve the degree of motoric fluency that was evident after 60 trials (Stöcker & Hoffmann, 2004) or 500+ trials per sequence (Verwey, 2003; Verwey & Eikelboom, 2003). Further research could examine whether increased practice reduces repetition costs or switching benefits when planning long sequences.

### Spatially compatible action effects and action sequence switching

In line with our prediction, spatially compatible (compared to incompatible) action effects *reduced* the sequence length effect, but specifically during sequence repetition (the sequence length effect was detected only in repetition trials). The results of Pilot Experiment 1 also suggested that compatibility reduced the sequence length effect (Appendix, Table 3, non-significant trend toward a Length-by-Compatibility interaction). We based this prediction on the notion that the ability to anticipate the spatially corresponding “visual” sequences may help to prime sequence retrieval (James, 1890; Koch, 2007; Shin et al., 2010) and thereby speed sequence initiation (Stöcker & Hoffmann, 2004). Compatibility may have reduced the sequence length effect in repetition trials by the same means: participants may have overcome increased planning demands of repeating long sequences by using the anticipated compatible visual effects to retrieve the correct sequences (Greenwald, 1972; Hommel et al., 2001). On the other hand, compatible action effects also increased error rates for long compared to short sequences. We speculate that compatible action-effect associations took time to learn, such that anticipated effects did not reliably prime accurate responses until later trials.

The observation that action-effect compatibility influenced initiation time (the time to lift the hand from the home position) but not start time (the time to press the first key of the current sequence) additionally supports a distinction between different processes that occur prior to execution (Diedrichsen & Kornysheva, 2015; Klapp, 1995; Rosenbaum et al., 1987; Verwey et al., 2015). Visual-spatial effect anticipation may have facilitated selection, enabling participants to retrieve the correct sequence in terms of its visual-spatial coordinates. This finding is consistent with previous proposals that stimulus-response compatibility and other features of movement outcomes are relevant to selecting the appropriate movement, i.e., retrieving and maintaining in short-term memory the perceptual or symbolic coordinates of the upcoming movement (e.g., Klapp, 1995; Verwey et al., 2015). Subsequent processes involved in programming the movement, such as specifying the movement parameters (e.g., Verwey et al., 2015), may have occurred after the perceptual coordinates were successfully retrieved, although evidence suggests that effect anticipation can also influence preparatory and execution processes that follow response selection (Kunde et al., 2004; Stöcker & Hoffmann, 2004).

## Conclusion

We here addressed how flexibly performers plan sequential actions when switching between multiple sequential actions. The results suggest that participants were slower to pre-plan longer sequences that they had just performed, but slower to execute long sequences after performing a different sequence. We hypothesize that performers may need to overcome interference between multiple chunks when repeating long sequences, and they may partially postpone planning until execution when switching. The findings suggest that planning and execution can be coordinated flexibly: performers may dynamically adjust whether planning takes place ahead of or during execution, based on current constraints. Furthermore, although task-switching behavior has been classically understood as costly, we here show evidence that repetition can be costly, and switching could be beneficial, when the task involves a longer action sequence that has been chunked into multiple planning units.

## Data Availability

The raw, anonymized datasets are available on psycharchives.org (10.23668/psycharchives.13143).

## Appendix

### Pilot experiments

Two pilot experiments were run to examine the influence of spatial action-effect compatibility on sequence length effects, and to explore the influence of action sequence switching on sequence length effects. We expected spatially compatible action effects to facilitate the planning of long sequences, and thus to reduce the initiation latency difference between long and short sequences (sequence length effect). We also expected action switching to slow the planning of long sequences, and thus to increase sequence length effects.

Note that the design of the pilot experiments was essentially the same as that of the main experiment, except that the number of switch and repeat trials was not uniform during the main task trials.

### Method

#### Participants

A total of 80 volunteers participated in the pilot experiments via Prolific: 35 in Pilot Experiment 1 and 45 in Pilot Experiment 2. Pre-screening criteria limited online recruitment to individuals who had upon registration with Prolific declared themselves to be between the ages of 18 and 35 years, right-handed, and to have normal or corrected-to-normal vision. After completing the study, participants were asked to verify their age, handedness, vision correction, and if they were neurologically healthy. Five participants were excluded from Pilot Experiment 1: two because they reported being left-handed or having uncorrected vision problems, and three because they did not report being neurologically healthy, yielding a final sample of 30, which included ten females, and a mean age of 22.7 years (*SD* = 4.3, range 18–34 years). One participant was excluded from Pilot Experiment 2 because they did not report being neurologically healthy, yielding a final sample of 44, including 14 females, and a mean age of 25.9 years (*SD* = 5, range 18–35 years). All participants provided informed consent, and the study was approved by the internal ethics committee at the Institute of Psychology at RWTH Aachen University and was conducted according to the Declaration of Helsinki.

#### Equipment and stimuli

Both pilot experiments were conducted online using Gorilla (gorilla.sc), with the same equipment requirements (laptop or desktop) as the main experiment. All stimuli were identical to those of the main experiment with the following exceptions.

Pilot Experiment 1 used two action sequences for all participants: one long sequence (M-O-K-O-K-M) and one short sequence (O-M-K). During the learning phase of the experiment, the words “bottom”, “middle”, or “top” were used as response cues for the “M”, “K”, and “O” keys, respectively. Some of the participants (*n* = 16) were shown the color cues and sequences in the learning task instructions, but this information was omitted from the instructions for remaining participants (*n* = 14) and for subsequent experiments, because we inferred that participants relied primarily on the learning trials rather than the instructions to learn the sequences. Learning accuracy was similar for both sets of participants.

Pilot Experiment 2 changed the spatial orientation of actions and effects from a vertical to a horizontal orientation. Participants pressed the “J”, “K”, and “L” keys with their index, middle, and ring finger, respectively, of their right hand, in four possible sequential orders: two possible long sequences (J-L-K-L-K-J, or L-J-K-J-K-L) or two possible short sequences (L-J-K, or J-L-K). The particular short and long action sequences that each participant performed was counterbalanced across participants, such that half of the participants performed J-L-K-L-K-J and L-J-K, and the other half performed L-J-K-J-K-L and J-L-K. During the learning phase of the experiment, the words “left”, “middle”, or “right” were used as response cues for the “J”, “K”, and “L” keys, respectively. During the main task, each key-press resulted in the appearance of a black square in one of three possible horizontal positions across the screen.

#### Procedure

All procedures were identical to those in the main experiment, with the exception that in Pilot Experiment 1 error feedback was presented for 500 ms, and in both Pilot experiments the number of switch and repeat trials was not uniform during the main task.

In addition, several changes were made to the procedure in Pilot Experiment 2. The duration of error feedback messages was lengthened to 2,500 ms, and a small amount of extra compensation was awarded for accurate performance, both in an effort to further encourage accuracy. In the main task, at the end of each block participants were awarded points based on their accuracy during the block (90% or greater = 1 point, 75% or greater = 0.5 points, less than 75% = 0 points). At the end of each block participants’ accuracy and points were displayed.

In Pilot Experiment 1, all participants performed with 94% or greater accuracy during Learning trials (*M* = 98.65%, *SD* = 1.09), and they performed each sequence correctly three times in succession after a mean of 3.9 trials per sequence during Practice trials (*SD* = 1.21, max = 8).

In Pilot Experiment 2, all participants performed with 90% or greater accuracy during Learning trials (*M* = 98.20%, *SD* = 1.88), and they performed each sequence correctly three times in succession after a mean of 4.25 trials per sequence during Practice trials (*SD* = 1.67, max = 9).

#### Design and analyses

All dependent variables were calculated and all analyses were run in the same way as in the main experiment.

### Results

#### Initiation time

##### Pilot Experiment 1

A main effect of Length and a marginal Length-by-Switch interaction (Table 3) suggested longer initiation times for long sequences compared to short sequences in Repeat trials but not Switch trials (see Fig. 4a). In Repeat trials, initiation time (ms) for long sequences trended toward exceeding that of short sequences (*long*: *M =* 546*, SE =* 32.9*,* 95%CI = [479, 614]; *short*: *M =* 514*, SE =* 25.1*,* 95%CI = [463, 566]; *difference* = 31.97, *SE =* 13.4, *t(29) =* 2.382, *p* = .1031, *g*_*rm*_* =* .16). In Switch trials, the difference between long and short sequences was smaller (*long*: *M =* 528*, SE =* 31.8*,* 95%CI = [463, 593]; *short*: *M =* 521*, SE =* 31.3*,* 95%CI = [457, 585]; *difference* = 7.37, *SE =* 10.3, *t(29) =* 0.712, *p* = .8914, *g*_*rm*_* =* .04).

In addition, a marginal Length-by-Compatibility interaction (Table 3) suggested longer initiation times for long sequences compared to short sequences in the incompatible condition, and less difference between long and short sequences in the compatible condition. In the incompatible condition, initiation time (ms) for long sequences marginally exceeded that of short sequences (*long*: *M =* 553*, SE =* 38.2*,* 95%CI = [475, 631]; *short*: *M =* 522*, SE =* 31.3*,* 95%CI = [458, 586]; *difference* = 30.55, *SE =* 12.66, *t(29) =* 2.413, *p* = .097, *g*_*rm*_* =* .14), and in the compatible condition the difference between long and short sequences was smaller (*long*: *M =* 522*, SE =* 28.8*,* 95%CI = [463, 580]; *short*: *M =* 513*, SE =* 26.3*,* 95%CI = [459, 567]; *difference* = 8.79, *SE =* 9.81, *t(29) =* 0.896, *p* = .8072, *g*_*rm*_* =* .06).

##### Pilot Experiment 2

A marginal main effect of Compatibility (Table 4) suggested longer initiation time (ms) in the incompatible condition (*M =* 526*, SE =* 22.1*,* 95%CI = [482, 571]) compared to the compatible condition (*M =* 504*, SE =* 18.5*,* 95%CI = [466, 541]).

#### Start time

##### Pilot Experiment 1

A Length-by-Switch interaction (Table 3) indicated longer start times for long sequences compared to short sequences in Repeat trials but not Switch trials (see Fig. 4b). In Repeat trials, start time (ms) for long sequences exceeded that of short sequences (*long*: *M =* 349*, SE =* 29.9*,* 95%CI = [288, 411]; *short*: *M =* 297*, SE =* 23.1*,* 95%CI = [250, 344]; *difference* = 52.6, *SE =* 15.1, *t(29) =* 3.48, *p* = .0083, *g*_*rm*_* =* .32). In Switch trials, the difference between long and short sequences was smaller (*long*: *M =* 309*, SE =* 28.3*,* 95%CI = [251, 367]; *short*: *M =* 327*, SE =* 26.0*,* 95%CI = [274, 380]; *difference* = 18.2, *SE =* 15.4, *t(29) =* 1.182, *p* = .6427, *g*_*rm*_* =* .12).

##### Pilot Experiment 2

A marginal main effect of Switch and a Length-by-Switch interaction (Table 4) indicated longer start times for long sequences compared to short sequences in Repeat trials, but not Switch trials (see Fig. 4e). In Repeat trials, start time (ms) for long sequences exceeded that of short sequences (*long*: *M =* 360*, SE =* 31.3*,* 95%CI = [297, 424]; *short*: *M =* 295*, SE =* 34.0*,* 95%CI = [226, 363]; *difference* = 65.6, *SE =* 19.2, *t(43) =* 3.417, *p* = .0073, *g*_*rm*_* =* .29). In Switch trials, the difference between long and short sequences was smaller (*long*: *M =* 294*, SE =* 27.8*,* 95%CI = [238, 350]; *short*: *M =* 328*, SE =* 36.7*,* 95%CI = [253, 402]; *difference* = 33.4, *SE =* 14.7, *t(43) =* 2.277, *p* = .1195, *g*_*rm*_* =* .13).

#### Mean inter-response time

##### Pilot Experiment 1

A main effect of Length (Table 3) indicated longer mean inter-response time (ms) for long sequences (*M* = 213, *SE* = 12.1, 95%CI = [188, 237]) compared to short sequences (*M* = 192, *SE* = 11.3, 95%CI = [168, 215]). A main effect of Switch indicated longer mean inter-response time for Repeat trials (*M* = 204, *SE* = 11.6, 95%CI = [180, 228]) than for Switch trials (*M* = 200, *SE* = 11.4, 95%CI = [177, 224]).

##### Pilot Experiment 2

A main effect of Length (Table 4) indicated longer mean inter-response time (ms) for long sequences (*M* = 245, *SE* = 12.3, 95%CI = [221, 270]) compared to short sequences (*M* = 222, *SE* = 12.7, 95%CI = [196, 248]). A main effect of Switch indicated longer mean inter-response time for Repeat trials (*M* = 236, *SE* = 12.3, 95%CI = [212, 261]) compared to Switch trials (*M* = 231, *SE* = 12.4, 95%CI = [206, 256]).

#### Error rate

##### Pilot Experiment 1

A main effect of Length (Table 3) indicated a higher error rate (percent incorrect trials) for long sequences (*M* = 8.67, *SE* = 1.22, 95%CI = [6.17, 11.16]) compared to short sequences (*M* = 6.09, *SE* = 0.93, 95%CI = [4.19, 7.99]). A main effect of Switch indicated a higher error rate for Repeat trials (*M* = 8.53, *SE* = 1.305, 95%CI = [5.86, 11.2]) compared to Switch trials (*M* = 6.23, *SE* = 0.868, 95%CI = [4.45, 8.0]).

##### Pilot Experiment 2

A main effect of Length (Table 4) indicated a higher error rate (percent incorrect trials) for long sequences (*M* = 7.9, *SE* = 0.942, 95%CI = [6.00, 9.80]) compared to short sequences (*M* = 4.4, *SE* = 0.53, 95%CI = [3.33, 5.47]). A marginal main effect of Switch suggested a higher error rate for Repeat trials (*M* = 6.72, *SE* = 0.789, 95%CI = [5.13, 8.31]) compared to Switch trials (*M* = 5.57, *SE* = 0.566, 95%CI = [4.43, 6.72]).

#### Inter-response time chunking

##### Pilot Experiment 1

We additionally explored whether spatial compatibility and action switching influenced inter-response time chunking by examining the chunk ratios in the first half of the long sequences and in the short sequences (start time divided by the mean of inter-responses 2–3). A 2 × 2 × 2 ANOVA (with the same factor structure as the analyses above) indicated a Length-by-Switch interaction (*F*(1,29) = 17.10, *MSE* = 0.10, *p* < .001, *η*^*2*^_*G*_ = .013, *η*^*2*^_*p*_ = .37). In Switch trials, the chunk ratio of short sequences exceeded that of long sequences (*short*: *M =* 1.71*, SE =* 0.136*,* 95%CI = [1.43, 1.98]; *long*: *M =* 1.42*, SE =* 0.13*,* 95%CI = [1.15, 1.69]; *difference* = 0.288, *SE =* 0.0749, *t(29) =* 3.837, *p* = .0033, *g*_*rm*_* =* .39). In Repeat trials, the difference between long and short sequences was smaller (*long*: *M =* 1.61*, SE =* 0.138*,* 95%CI = [1.32, 1.89]; *short*: *M =* 1.55*, SE =* 0.12*,* 95%CI = [1.3, 1.8]; *difference* = 0.057, *SE =* 0.0681, *t(29)=* 0.837, *p* = .8366, *g*_*rm*_* =* .08) (see Fig. 4c). No other statistically significant effects were found.

##### Pilot Experiment 2

A 2 × 2 × 2 ANOVA on mean chunk ratio in the first half of the long sequences compared to that of the short sequences indicated a Length-by-Switch interaction (*F*(1,43) = 24.77, *MSE* = 0.15, *p* < .001, *η*^*2*^_*G*_ = .012, *η*^*2*^_*p*_ = .37). In Switch trials, the chunk ratio of short sequences exceeded that of long sequences (*short*: *M =* 1.48*, SE =* 0.166*,* 95%CI = [1.141, 1.81]; *long*: *M =* 1.18*, SE =* 0.112*,* 95%CI = [0.959, 1.41]; *difference* = 0.291, *SE =* 0.073, *t(43) =* 3.984, *p* = .0014, *g*_*rm*_* =* .22). In Repeat trials, the difference between long and short sequences was smaller (*long*: *M =* 1.45*, SE =* 0.126*,* 95%CI = [1.197, 1.71]; *short*: *M =* 1.33*, SE =* 0.153*,* 95%CI = [1.018, 1.64]; *difference* = 0.124, *SE =* 0.0854, *t(43)=* 1.447, *p* = .4777, *g*_*rm*_* =* .12) (see Fig. 4f). No other statistically significant effects were found.

**Table 3:** Pilot Experiment 1 ANOVA summary table per dependent variable

Initiation time	Source	*df*	MSE	*F*	*η*^*2*^_*G*_	*η*^*2*^_*p*_	*p*
	Length	1,29	5538.89	4.19	.003	.13	.050
	Switch	1,29	3720.48	0.58	<.001	.02	.454
	Compatibility	1,29	19953.00	1.24	.003	.04	.274
	Length × Switch	1,29	3071.18	2.96	.001	.09	.096
	Length × Compatibility	1,29	2159.17	3.29	<.001	.10	.080
	Switch × Compatibility	1,29	2119.65	0.00	<.001	.00	.966
	Length × Switch × Compatibility	1,29	2632.95	0.09	<.001	.00	.760
Start time	Source	*df*	MSE	*F*	*η*^*2*^_*G*_	*η*^*2*^_*p*_	*p*
	Length	1,29	9498.04	1.86	.003	.06	.183
	Switch	1,29	5108.53	0.31	<.001	.01	.583
	Compatibility	1,29	14878.67	1.38	.004	.05	.250
	Length × Switch	1,29	4493.94	16.74	.013	.37	<.001
	Length × Compatibility	1,29	2096.21	0.38	<.001	.01	.540
	Switch × Compatibility	1,29	2749.87	1.02	<.001	.03	.321
	Length × Switch × Compatibility	1,29	2138.53	1.40	<.001	.05	.246
Inter-response time	Source	*df*	MSE	*F*	*η*^*2*^_*G*_	*η*^*2*^_*p*_	*p*
	Length	1,29	1159.88	22.98	.025	.44	<.001
	Switch	1,29	110.94	6.95	<.001	.19	.013
	Compatibility	1,29	2045.13	0.36	<.001	.01	.553
	Length × Switch	1,29	114.57	0.05	<.001	.00	.833
	Length × Compatibility	1,29	194.59	2.25	<.001	.07	.144
	Switch × Compatibility	1,29	182.32	1.17	<.001	.04	.288
	Length × Switch × Compatibility	1,29	93.69	1.71	<.001	.06	.201
Error rate	Source	*df*	MSE	*F*	*η*^*2*^_*G*_	*η*^*2*^_*p*_	*p*
	Length	1,29	50.78	7.83	.024	.21	.009
	Switch	1,29	63.57	5.01	.019	.15	.033
	Compatibility	1,29	63.14	0.00	<.001	.00	.972
	Length × Switch	1,29	48.04	0.93	.003	.03	.343
	Length × Compatibility	1,29	44.16	1.39	.004	.05	.248
	Switch × Compatibility	1,29	34.09	0.73	.002	.02	.399
	Length × Switch × Compatibility	1,29	21.41	1.75	.002	.06	.197

*MSE* mean square error

**Table 4:** Pilot Experiment 2 ANOVA summary table per dependent variable

Initiation time	Source	*df*	MSE	*F*	*η*^*2*^_*G*_	*η*^*2*^_*p*_	*p*
	Length	1, 43	3342.73	0.21	<.001	.01	.652
	Switch	1, 43	4375.88	0.09	<.001	.00	.765
	Compatibility	1, 43	12709.22	3.58	.006	.08	.065
	Length × Switch	1, 43	4105.72	0.57	<.001	.01	.453
	Length × Compatibility	1, 43	3443.81	0.00	<.001	.00	.960
	Switch × Compatibility	1, 43	1279.24	0.18	<.001	.00	.675
	Length × Switch × Compatibility	1, 43	3001.46	0.01	<.001	.00	.942
Start time	Source	*df*	MSE	*F*	*η*^*2*^_*G*_	*η*^*2*^_*p*_	*p*
	Length	1, 43	16995.96	1.34	.001	.03	.254
	Switch	1, 43	8413.42	2.93	.001	.06	.094
	Compatibility	1, 43	18679.01	0.19	<.001	.00	.664
	Length × Switch	1, 43	8701.51	24.79	.012	.37	<.001
	Length × Compatibility	1, 43	3766.52	0.07	<.001	.00	.790
	Switch × Compatibility	1, 43	5012.27	0.57	<.001	.01	.454
	Length × Switch × Compatibility	1, 43	3355.10	1.97	<.001	.04	.168
Inter-response time	Source	*df*	MSE	*F*	*η*^*2*^_*G*_	*η*^*2*^_*p*_	*p*
	Length	1, 43	1621.14	29.96	.019	.41	<.001
	Switch	1, 43	223.87	10.75	<.001	.20	.002
	Compatibility	1, 43	2083.43	0.62	<.001	.01	.436
	Length × Switch	1, 43	235.32	0.17	<.001	.00	.685
	Length × Compatibility	1, 43	416.85	1.56	<.001	.04	.218
	Switch × Compatibility	1, 43	193.34	2.21	<.001	.05	.144
	Length × Switch × Compatibility	1, 43	241.53	0.00	<.001	.00	.953
Error rate	Source	*df*	MSE	*F*	*η*^*2*^_*G*_	*η*^*2*^_*p*_	*p*
	Length	1, 43	73.79	14.60	.052	.25	<.001
	Switch	1, 43	34.12	3.42	.006	.07	.071
	Compatibility	1, 43	55.98	0.17	<.001	.00	.684
	Length × Switch	1, 43	39.82	0.65	.001	.01	.424
	Length × Compatibility	1, 43	45.10	1.43	.003	.03	.238
	Switch × Compatibility	1, 43	41.96	0.78	.002	.02	.382
	Length × Switch × Compatibility	1, 43	34.34	2.61	.005	.06	.114

*MSE* mean square error

## Code availability

The custom R code used for the current study is available on psycharchives.org (10.23668/psycharchives.13144).
